# Moroccan Experience of Targeted Screening for Inborn Errors of Metabolism by Tandem Mass Spectrometry

**DOI:** 10.3390/pediatric15010018

**Published:** 2023-03-10

**Authors:** Faïza Meiouet, Sâad El Kabbaj, Rachid Abilkassem, François Boemer

**Affiliations:** 1 Laboratoire de Recherche et d’Analyses Médicales de la Gendarmerie Royale, Avenue Ibn Sina, Agdal, Rabat 10100, Morocco; 2 Service de Pédiatrie, Hôpital Militaire d’Instruction Mohamed V, Rabat 10100, Morocco; 3 Laboratoire de Biochimie Génétique, Centre de Maladies Métaboliques, CHU Sart-Tilman, CHU Liège, 4000 Liege, Belgium

**Keywords:** tandem mass spectrometry, inborn errors of metabolism, amino acid disorders, fatty acid oxidation disorders, organic acidemias, Morocco

## Abstract

Background: Expanded newborn screening using tandem mass spectrometry (MS/MS) for inborn errors of metabolism (IEM), such as organic acidemias (OAs), fatty acid oxidation disorders (FAODs), and amino acid disorders (AAs), is increasingly popular but has not yet been introduced in Africa. With this study, we aim to establish the disease spectrum and frequency of inborn errors of OAs, FAODs, and AAs in Morocco. Methods: Selective screening was performed among infants and children suspected to be affected with IEM between 2016 and 2021. Amino acids and acylcarnitines spotted on filter paper were analyzed using MS/MS. Results: Out of 1178 patients with a clinical suspicion, 137 (11.62%) were diagnosed with IEM, of which 121 (88.3%) patients suffered from amino acids disorders, 11 (8%) were affected by FAOD, and 5 (3.7%) by an OA. Conclusions: This study shows that various types of IEM are also present in Morocco. Furthermore, MS/MS is an indispensable tool for early diagnosis and management of this group of disorders.

## 1. Introduction

Newborn screening (NBS) implemented by Dr. Robert Guthrie [1,2] for the diagnosis of phenylketonuria (PKU) has evolved continuously since the 1960s, especially thanks to the introduction of screening methods using dried blood spot (DBS) samples [3]. To date, several countries around the world have established national NS programs, based both on the Wilson and Jungner criteria and on specific screening policies [4,5,6].

Initially, NBS began with the detection of congenital hypothyroidism (HC) and PKU [1,7]. From the 1990s, when tandem mass spectrometry (MS/MS) was introduced into NS laboratories, it became possible to screen simultaneously for a large number of disorders [8,9,10]. Therefore, MS/MS became a tool for the detection and prevention of rare and serious diseases that can lead to permanent disability or sometimes premature death. Since the early 2000s, several developed countries have been equipped with this technology for clinical diagnosis and have extended their NBS program to more than 40 inborn errors of metabolism (IEM) [11,12,13,14]. This screening technology has enabled the identification of amino acid disorders (AAs), fatty acid oxidation disorders (FAODs), and organic acidemia (OAs) with a specificity of up to 99.90% for each of the AAs and FAODs, and 99.87% for the OAs [12,13].

These IEM are genetic disorders, most often with autosomal recessive inheritance. They appear during infancy and more generally in early childhood, and their incidence varies according to race and ethnicity with predominance in populations where consanguineous marriages are common. An early diagnosis and treatment established promptly can avoid the development of severe consequences such as neurological lesions [15,16,17].

In Morocco, NBS for IEM is not institutionalized yet. Therefore, the incidence of these anomalies is unknown. The clinical presentations of IEM are however often not specific, and these disorders are usually underdiagnosed. With this research study, we sought to assess retrospectively the diagnostic contribution of selected biomarkers (amino acids and acylcarnitines) on DBS to detect IEM in our country by MS/MS.

## 2. Materials and Methods

### 2.1. Study Population

An observational study was conducted based on the results of metabolic analyses of infants or children referred to the Laboratoire de Recherche et d’Analyses Médicales de la Gendarmerie Royale (LRAM) in Rabat between February 2016 and February 2021. MS/MS analysis was requested by pediatricians when an IEM was suspected based on family history or on clinical suggestive settings, such as: lethargy, coma, seizures, vomiting in a neonate, hyperammonemia, metabolic acidosis or respiratory alkalosis, lactic acidemia, unexplained developmental delay or intellectual disability. 

For all the patients referred to our laboratory, amino acid and acylcarnitines profiles were studied using the “MassChrom^®^ Amino acids and Acylcarnitines from dried blood” kit (Chromsystems, Munchen, Germany). Our laboratory opted to run analysis on DBS specimens as sampling can be carried out outside the healthcare structure by a simple capillary puncture. In addition to the advantage of exhibiting excellent stability over time, DBS samples provide incomparable logistical ease. This type of sample can indeed be easily shipped by post, thus potentially offering access to the diagnosis of IEM to the entire Moroccan territory.

### 2.2. Sample Preparation

Acylcarnitines and amino acids were extracted from DBS samples according to the manufacturer’s recommendations. To a 3.2 mm disk punched into a 96-well microtiter filter plate was added 200 µL of extraction buffer containing the following labeled internal standards: [D_9_]carnitine, [D_3_]acetylcarnitine, [D_3_]propionylcarnitine, [D_3_]butyrylcarnitine, [D_6_]glutarylcarnitine, [D_9_]isovalerylcarnitine, [D_3_]hexanoylcarnitine, [D_3_]octanoylcarnitine, [D_3_]decanoylcarnitine, [D_3_]dodecanoylcarnitine, [D_3_]tetradecanoylcarnitine, [D_3_]hexadecanoylcarnitine, [D_3_]octadecanoylcarnitine, [D_4_]alanine, [D_8_]valine, [D_3_]leucine, [D_3_]methionine, [D_5_]phenylalanine, [D_4_]tyrosine, [D_3_]aspartate, [D_5_]glutamate, [D_6_]ornithine, [D_7_]arginine, [D_2_]citrulline, [^15^N,^13^C_2_]glycine), [D_7_]proline. After 20 min of mixing, the samples were centrifuged, and the eluate was evaporated under a nitrogen stream, reconstituted with 60 µL of derivatization solution (butanol/acetylchloride), and incubated for 15 min at 65 °C. Butylated samples were again vaporized under a nitrogen stream, and the residue was reconstituted into 100 µL of reconstitution solution. The extracts were then ready to be injected into the mass spectrometer. The entire extraction step was duplicated for each patient from the same filter paper to guarantee reliable results.

### 2.3. Amino Acids and Acylcarnitines Profile and Mass Spectrometry

A rapid method for the simultaneous detection and quantification of butylated acylcarnitines and amino acids was performed on a triple quadrupole UPLC XEVO TQD IVD System (Waters, Milford, MA, USA). 20 µL of each sample were injected with a total run time equal to 1.6 min. All the measurements were carried out in the positive ion electrospray mode and using multiple reaction monitoring (MRM). Data processing and determination of concentrations were performed using MassLynx version 4.1 software by calculating the ratio between the area of each compound and the surface of its corresponding internal standard. 

The 47 analytes quantified for IEM screening were the following: 13 amino acids: alanine (Ala), arginine (Arg), aspartate (Asp), citrulline (Cit), glutamate (Glu), glycine (Gly), leucine/isoleucine (Leu/Iso), methionine (Met), ornithine (Orn), phenylalanine (Phe), proline (Pro), tyrosine (Tyr), valine (Val), free carnitine (C0) and 33 acylcarnitines: acetylcarnitine (C2), propionylcarnitine (C3), iso-/butyrylcarnitine (C4), isovaleryl-/2-methylbutyrylcarnitine (C5), glutarylcarnitine (C5DC), hexanoylcarnitine (C6), octanoylcarnitine (C8), decanoylcarnitine (C10), dodecanoylcarnitine (C12), tetradecanoylcarnitine (C14), hexadecanoylcarnitine (C16), octadecanoylcarnitine (C18), tiglylcarnitine (C5:1), 3-hydroxy-isovalerylcarnitine (C5OH), octenoylcarnitine (C8:1), malonylcarnitine (C3DC), decadienoylcarnitine (C10:2), decenoylcarnitine (C10:1), methylmalonyl-/succinylcarnitine (C4DC), dodecenoylcarnitine (C12:1), 3-methylglutarylcarnitine (C6DC), tetradecadienoylcarnitine (C14:2), tetradecenoylcarnitine (C14:1), octanedioylcarnitine (C8DC), 3-hydroxy-tetradecanoylcarnitine (C14OH), hexadecenoylcarnitine (C16:1), sebacylcarnitine (C10DC), 3-hydroxy-hexadecenoylcarnitine (C16:1-OH), 3-hydroxy-hexadecanoylcarnitine (C16OH), octadecadienoylcarnitine (C18:2), octadecenoylcarnitine (C18:1), 3-hydroxy-octadecadienoylcarnitine (C18:2-OH), 3-hydroxy-octadecenoylcarnitine (C18:1-OH). When a specific stable isotope was not available, the following ratios were used for quantitation: C4DC/C4D3, C5:1/C5-D9, C5OH/C5-D9, C8:1/C8D3, C10:1/C10D3, C10:2/C10D3, C14:1/C14D3, C14:2/C14D3, C14OH/C14D3, C16:1/C16-D3, C16:1-OH/C16-D3, C16OH/C16-D3, C18:1/C18D3, C18:2/C18D3, C18:1OH/C18D3, C18:2OH/C18D3.

### 2.4. Decision Criteria for Interpretation

For each parameter, analytical interpretation of the results was based on reference values established between the 0.5th and 99.5th centiles of a healthy population. A total of 8 acylcarnitine ratios and 47 metabolite concentrations were compared with their respective cut-off values. Any result falling outside its reference range was systematically retested. Decision criteria for AA disorders, OAs, and FAODs are listed in Table 1 and Table 2. Confirmation of IEM diagnosis was established by a clinical examination and a study of urinary organic acids by GC-MS. The main results were expressed as means ± standard deviation (SD). For phenylketonuria, specifically, the results were expressed according to the intergroup comparison and a Student’s test. A result was considered as statistically significant when the *p*-value was lower than 0.05.

## 3. Results

When setting up the method for determining amino acids and acylcarnitines by MS/MS, we configured the acquisition of all the biomarkers mentioned above on our mass spectrometer to identify approximately 31 IEMs.

From 1178 symptomatic patients screened, 137 (11.62%) were assigned to an IEM, reflecting the high diagnostic yield of the method. A total of 71 were females and 66 were males. The average age of the patients screened was 4.9 ± 5.8 years. There were 121 (88.3%) patients with amino acid disorders, 11 (8%) with fatty acid oxidation, and 5 (3.7%) with organic acidemias. In 101 cases (73.8%), parental consanguinity was established of which 60 (59.4%) were of first degree, and 41 (40.6%) of second degree. An unexplained death was found in many families.

### 3.1. Amino Acid Disorders

Targeted screening by MS/MS yielded a high frequency of aminoacidopathies, with phenylketonuria being the most common abnormality observed in 104 (86%) of 121 patients with AA disorders. A concentration of 2450 µmol/L, exceeding 20-fold the normal level (reference value < 120 µmol/L) was measured in a 3-year-old girl. A case of Dihydropterine Reducase (DHPR) deficiency was observed in a 4-year-old girl with hyperphenylalaninaemia (phenylalanine = 769 µmol/L). Molecular analysis of the *QPDR* gene confirmed the DHPR deficiency.

Hyperthyrosinemia was discovered in seven cases with an average concentration of 389.6 µmol/L, more than six-fold higher than a normal concentration (60 µmol/L). The age of affected patients ranged from 1 to 7 years with similar clinical symptoms (hepatomegaly, abnormal coagulation, renal dysfunction, and anorexia). Extremely elevated excretions of 4-hydroxyphenyl-lactate and 4-hydroxyphenyl-pyruvate in urine were present in all patients. The succinylacetone detected in urine helped in confirming the diagnosis.

Five patients with neurological abnormalities were diagnosed with cystathionine β-synthase deficiency (CBS) deficiency, using methionine as a biomarker. The average methionine concentration in the five patients was 455.7 ± 333.5 µmol/L, and high methionine levels exceeding 800 µmol/L were observed in a 7-year-old girl. The determination of plasma total homocysteine (tHcy) showed hyperhomocysteinemia in all cases, confirming the diagnosis.

Two patients were identified with a massive elevation of leu/iso (2352 µmol/L and 1321.6 µmol/L), at 7 and 21 days, respectively. They were classified as classic maple syrup urine disease (MSUD), whose clinical signs were accompanied by a severe neonatal-onset form with neurological distress, coma, and death. Concomitantly with the onset of the symptoms, the patients emitted an intense maple syrup-like odor. The clinical findings and the high concentrations of branched amino acids, mainly leu/iso and valine, were perfectly correlated for the diagnosis of MSUD.

One case of suspected nonketotic hyperglycinemia (NKH) was observed with severe clinical symptoms, profound coma, and apnea. The NKH was suspected following the simultaneous increase in elevated glycine in DBS: 695 µmol/L (normal values < 417 µmol/L), in plasma: 432 µmol/L (normal values < 291 µmol/L), and in cerebrospinal fluid (CSF): 14 µmol/L (normal values < 8.4 µmol/L) with a ratio CSF glycine/plasma glycine: 0.03 (normal values: 0.02–0.03). The patient died after 6 days of life. A few years ago, her brother died with similar symptoms at birth. 

### 3.2. Urea Cycle Disorders

Among the urea cycle disorders, two cases of hyperargininemia were observed. One of them presented arginine levels at 359 µmol/L, exceeding 15-fold the normal level (reference values < 56.5 µmol/L).

### 3.3. Organic Acidemias

The profile of acylcarnitines plays a key role in the diagnosis of IEM, in particular for the diagnosis of FAODs and OAs. During our study, we detected five organic acidemia cases (3.7%) mainly by the following biomarkers: C3, C5DC, and also with the ratio C3/C0. In three cases, C3 was increased with a normal concentration of C4DC. These results were very suggestive of propionic acidemia (PA) or methylmalonic acidemia (MMA), or vitamin B12 deficiency. In one case, C3 was 10 times greater (13.5 µmol/L) than the normal range (<2.46 µmol/L), and also the C3/C0 ratio was 10 times greater than the reference interval (0.02–0.08). These abnormalities were accompanied by an increase in glycine as well.

Glutaric aciduria type I (GA1) was diagnosed in two male infants with increased C5DC. In one case, the C5DC concentration reached 4 µmol/L (normal values < 0.49 µmol/L), exceeding 14 times the normal level. The diagnosis was confirmed in one case by the evaluation of urinary organic acids, which revealed an increase in glutaric and 3-hydroxyglutaric acids. In the second case, the molecular genetic analysis of the *GCDH* gene demonstrated the presence of the homozygous pathogenic variant c.12113A > G (p.Met405Val).

### 3.4. Fatty Acid Oxidation Disorders

A free carnitine (C0) level below 5 µmol/L was used as a threshold to identify carnitine deficiencies. In our study, we identified 11 cases (7 males and 4 females) in which the levels of C0 varied between 1.66 and 5 µmol/L with an average concentration of 4.19 ± 1.13 µmol/L. Similarly, in 30 cases with diagnosed IEMs, we observed low levels of free carnitine (6–10 µmol/L) secondary to homocystinuria, argininemia, OTC deficiency, or pharmacological treatment with valproate.

## 4. Discussion

This prospective study targeting the diagnosis of hereditary intermediate metabolism diseases by MS/MS identified 9 IEMs among the 31 searched. Although this number is underestimated and does not reflect reality, it highlights the high frequency of certain IEMs in Morocco, which can guide decision-making for the establishment of a national NBS program. In fact, Morocco has undertaken several efforts to adopt the new National Integrated Child Health Policy (UNICEF 2019) and has made significant progress in child health. Morocco’s action plans aim to strengthen a high-quality equitable health system and to provide universal health coverage. In 2006, the Ministry of Health hosted in Marrakech the first MENA regional NBS conference entitled: “Strengthening Newborn Screening in the Middle East and North Africa”, where screening for congenital hypothyroidism (CH) was strongly recommended [18]. This decision was integrated into the national action plan of the Moroccan Ministry of Health (health plan 2025) after various regional pilot studies [19,20]. The integration of screening for phenylketonuria is part of the action plan of the Ministry of Health. Prerequisites for the implementation of the program are being developed.

Even if the interest of NBS is obvious for health professionals in Morocco, the diagnosis of IEM by tandem mass spectrometry faces economic and technological difficulties due to the elevated cost of the technology and of dedicated reagents, the lack of a service provider specialized in the maintenance of these instruments, and also the lack of trained personnel in the use of this technology in a medical environment. This study revealed an overall positivity rate of 11.6% of IEM in our patients, showing that aminoacidopathies, organic acidemias, and fatty acid oxidation disorders are not rare in Morocco. A frequency of 10.8% of IEM has been reported in previous studies carried out in Oman [21] on severe pediatric cases, while in China, Han. L et al. observed a proportion of 6.2% of metabolic abnormalities in suspected patients [22]. Furthermore, positivity rates of 2.8% and 0.53% of IEM cases were reported from Tunisia [23] and Libya [24], our neighboring countries in the Maghreb. These results show a wide range of positivity rates in the detection of IEM, revealing a greater prevalence of these anomalies in certain geographical areas due to the patient selection criteria and also to the screening and diagnostic strategy.

Aminoacidopathies are anomalies that result in intoxication, secondary to the accumulation of toxic metabolites upstream of an enzymatic block. The clinical diagnosis is primarily biochemical, and the analysis of amino acids is sufficiently informative to obtain a diagnosis [25]. In the study population, amino acid disorders accounted for 88.3% of all abnormalities, with a predominant frequency of phenylketonuria occurring in 104 (86%) of all 121 amino acids cases with a late age of diagnosis, achieved beyond 9 months in 90.4% of cases. According to our results, classic and moderate phenylketonuria is mainly diagnosed in our population in both sexes. The median phenylalanine concentration is equal to 1107 µmol/L; this rate is 26 times higher than that found in the control group (median value = 42 µmol/L) (*p* < 0.0001). All of the phenylketonuric patients have a high ratio Phe/Tyr: 38 ± 19.1 µmol/L and 44.5 ± 24.3 µmol/L in female and male, respectively, vs. the mean in the control group: 0.8 ± 0.2 µmol/L and 0.9 ± 0.2 µmol/L in female and male, respectively. In this phenylketonuric group, the median concentration of tyrosine was 27 µmol/L vs. 48 µmol/L in the control group (*p* < 0.0001). Only a small proportion (9.6%), referred on the basis of an index case in the family, could be diagnosed early in an age interval between 3 days and 2 months. Confirmation of the diagnosis is achieved by checking the concentrations of phenylalanine on a new DBS sample and by molecular analysis of the *PAH* gene. To rule out any possible (DHPR) deficiency, measurement of urinary biopterins and blood DHPR activity should also be considered. If this analysis is positive, analysis of the *QDPR* gene should be considered [26]. 

Depending on geographical areas and ethnic groups, there are wide variations in the incidence of phenylketonuria. It is approximately 1:10,000 in European populations with a very high incidence in Ireland (1:4500) and lower in Finland (1:200,000) [27]. In Turkey, the incidence is also high (1:2600) [27], followed by Tunisia (1:7631) [28].

These countries share with Morocco the same culture and customs, including consanguineous marriages, which could be one of the causes of recessive IEM such as phenylketonuria. Overall, a similar trend was observed in some countries that carried out selective screening such as Oman [21], Tunisia [23], and India [29]. Only 5% of tyrosinaemia was detected during the selective screening, although the rate observed for tyrosinemia remains relatively low and does not reflect its real frequency. Our results revealed a difference in detection compared to certain Asian countries, in particular Japan, where no case of tyrosinemia was observed [30]. 

FAODs are characterised by clinical presentations such as acute hypoketotic hypoglycemia, cardiomyopathy, and myopathy. Some patients may be suffering from only one of these symptoms, whereas others can present with all three phenotypes, depending on the residual enzyme activity, the age of the patient, and exposure to stress; hence the need to identify these anomalies early for better management [17]. Several countries use free carnitine as a marker of primary carnitine deficiency (PCD) when the C0 concentration is <5 µmol/L in dried blood spot (DBS) samples (normal values: 25–50 µmol/L). If the results suggest a specific diagnosis, this is confirmed by enzyme assays or mutation analysis of the *SLC22A5* gene [31]. In our experience, the 11 cases observed with C0 deficiency could be due to primary carnitine deficiency or subsequent to maternal carnitine deficiency. In the other 30 cases of screened patients, the low C0 level was secondary to an IEM, chronic renal failure, malnutrition, and also valproate or antibiotic treatment. Currently, there are few data on the incidence of primary carnitine deficiency. It seems that it is in Japan [30] that PCD is more frequent, with an incidence of 1 in 40,000.

It emerges from this study that most FAODs, in particular MCAD, VLCAD, LCHAD, SCAD, CACT, CPT I, and CPT II seem to be very rare in our population. Despite the recommendations of our laboratory regarding sampling conditions (i.e., fasting for 14 h for a young child, fasting for 4 to 6 h after breastfeeding for a newborn, and also at a distance from a glucose infusion), we cannot state whether this rarity is related to non-compliance to these pre-analytical constraints by parents when collecting blood, or to the low prevalence of these disorders in the Moroccan population. Only a nationwide implementation of studies such as a formal NBS program could provide clear insights.

This targeted screening enabled the diagnosis of two cases of GA I and only three cases of MMA/PA despite a low cut-off (2.46 µmol/L) for interpreting the results of propionylcarnitine (C3). Our results are in agreement with Dingabur’s study on the South Indian population where MMA, PA, and GA I are the most detected organic aciduries [29]. In MMA/PA disorders, the cut-off value is very important to define because it can generate false positives or false negatives. The Pajares [32] study highlighted an increase in cases of newborns with disturbed MMA/PA markers from 1.18% to 3.8% when the C3 cut-off was reduced from 4.5 µmol/L to 3.5 µmol/L. However, the increase in C3 and the C3/C2 ratio makes it possible to initiate additional investigations in order to identify anomalies of genetic origin from those acquired, in particular, vitamin B12 deficiency in newborns due to malnutrition or a vegetarian diet from the mother.

The evaluation of the clinical information from the population screened for IEM in this study revealed a significant proportion of newborns and children are from consanguineous marriages in 73.8%, unexplained deaths in the siblings, as well as a high number of families with the siblings affected by phenylketonuria. This is in agreement with the results of a Moroccan study carried out among 176 families suffering from autosomal recessive diseases of which 59% of the cases are due to consanguinity. The most common type of consanguineous marriage was between first cousins, with 58.5% of total consanguineous marriages and 8.9% of all marriages [33].

Several studies have reported the impact of consanguineous marriages on the rate of infant mortality and hereditary metabolic diseases. Shulpen’s study in the Netherlands found a 4-5 times higher proportion of deaths from hereditary causes in the Moroccan and Turkish population compared to Surinamese/Antillians and indigenous Dutch [34]. Similarly, a recent study covering a period of 27 years in the south of Israel revealed a high incidence of IEM in children of Bedouin-Muslim origin compared to those of Jewish origin: 101.6/100,000 vs. 16/100,000, respectively [35]. In addition, a Danish study carried out to evaluate the impact of consanguinity on the frequency of IEM according to ethnic groups and countries, found an overall frequency 25.5 times higher as well as a high incidence of IEM in children from Arab, Afghan, Turkish, and Pakistani populations in comparison with children of Danish origin (5.35:10,000 vs. 0.21:10,000) [36].

## 5. Conclusions

In epidemiological terms, the diagnostic yield of the profile of amino acids and acylcarnitines by tandem mass spectrometry seems relatively accurate for symptomatic IEM. This retrospective study at LRAM shows that different types of IEM are also prevalent in Moroccan communities. Their early identification is essential to avoid irreversible sequels with appropriate treatment. With this study, we hope for the development of a national NBS program as well as prevention programs similar to those already in place in developed countries.

## Figures and Tables

**Table 1:** Biomarker cut-offs for amino acid disorders.

Disorder	Cut Off (µmol/L)	Cut Off Molar Ratio
Phenylketonuria Hyperphenylalaninemia	Phe > 120	Phe/Tyr > 3
Tyrosinemia type 1	Tyr > 127.1 Succinylacetone > 1	
Tyrosinemia type 2	Tyr > 127.1	
Tyrosinemia type 3	Tyr > 127.1	
Citrullinemia type 1	Cit > 38.5	
Hyperprolinemia	Pro > 274.1	
Hyperornithinemia	Orn > 142.3	
Hypermethioninemia	Met > 27.3	
Maple syrup urine disease	Leu/iso > 223.74	
Nonketotic hyperglycinemia	DBS: Gly > 417.7 Plasma: Gly > 291 Cerebrospinal fluid (CSF): Gly > 8.4	Gly CSF/Gly plasma > 0.03
Homocystinuria	Met > 27.3	
Argininemia	Arg > 56.5	

**Table 2:** Biomarker cut-offs for OAs and FAODs.

Disorder	Cut Off Marker (µmol/L)	Cut Off Marker Molar Ratio
Propionic aciduria (PA)	C3 > 2.46	C3/C0 > 0.08 C3/C2 > 0.14
Methylmalonic aciduria (MMA)	C3 > 2.46 C4DC > 0.86	C3/C0 > 0.08 C3/C2 > 0.14
Glutaric aciduria type 1 (GA I)	C5DC > 0.45	
Glutaric aciduria type 2 (GA II)	C4 > 0.94 C5 > 0.33	
Isovaleric aciduria (IVA)	C5 > 0.33	
3-Hydroxy-3-methylglutaryl-CoA lyase deficiency (HMG)	C5OH > 0.5 C6DC > 0.1	
β-ketothiolase deficiency (BKT)	C5:1 > 0.07	
3-methylcrotonyl-CoA carboxylase deficiency (3-MCC)	C5OH > 0.51	
3-methyl glutaconic aciduria (3-MGA)	C5OH > 0.51	
Ethylmalonic encephalopathy (EE)	C4 > 0.94 C5 > 0.33	
Malonic aciduria	C3DC > 0.15	
Medium chain acyl-CoA dehydrogenase deficiency (MCAD)	C8 > 0.24 C6 > 0.14 C10:1 > 0.49	C8/C2 > 0.01 C8/C10 > 2.17
Very long chain acyl-CoA dehydrogenase deficiency (VLCAD)	C14:1 > 0.18 C12:1 > 0.13 C12 > 0.22 C14:2 > 0.08 C14 > 0.37	C14:1/C12:1 > 4.53
Long chain hydroxy acyl-CoA dehydrogenase (LCHAD)	C16-OH > 0.08 C18:2-OH > 0.06 C18:1-OH > 0.07 C14-OH > 0.06 C16:1-OH > 0.12	
Carnitine palmitoyltransferase deficiency type 1 (CPT 1)	C0 > 52.98 C16 < 0.38 C18 < 0.23	C0/C16 + C18 > 37.48
Carnitine palmitoyltransferase deficiency type 2 (CPT 2)	C18 > 1.18 C18:2 > 0.8 C18:1 > 1.92 C16:1 > 0.4 C16 > 2.94 C0 < 13.63	C16+ C18:1/C2 > 0.27
Carnitine-acylcarnitine translocase deficiency (CACT)	C16 > 2.94 C18:1 > 1.92 C18:2 > 0.8 C18 > 1.18 C0 < 13.63	
Carnitine uptake defect (CUD)/Primary carnitine deficiency (PCD)	C0 < 5	
Short chain acyl-CoA dehydrogenase deficiency (SCAD)	C4 > 0.94	

## Data Availability

The data presented in this study are available on request from the corresponding author.
